# Effect of crucible wall roughness on the laminar/turbulent flow transition of the Ga75In25 alloy stirred by a rotating magnetic field

**DOI:** 10.1038/s41598-022-21898-7

**Published:** 2022-11-03

**Authors:** András Roósz, Arnold Rónaföldi, Mária Svéda, Zsolt Veres

**Affiliations:** 1 Institute of Physical Metallurgy, Metalforming and Nanotechnology, University of Miskolc, Miskolc, 3515 Hungary; 2 ELKH-ME Materials Science Research Group, Miskolc, 3515 Hungary

**Keywords:** Engineering, Materials science

## Abstract

The critical magnetic induction (*Bcr*) values of a melt flow produced by a rotating magnetic field (RMF), remaining laminar or turbulent, are essential in different solidification processes. In an earlier paper (Metall Res Technol 100: 1043–1061, 2003), we showed that *Bcr* depends on the crucible radius (*R*) and frequency of the magnetic field (*f*). The effect of wall roughness (*WR*) on *Bcr* was investigated in this study. Using ten different wall materials, we determined the angular frequency (ω) and Reynolds number (*Re*) as a function of the magnetic induction (*B*) and *f* using two different measuring methods (pressure compensation method, PCM; height measuring method, HMM). The experiments were performed at room temperature; therefore, the Ga75wt%In25wt% alloy was chosen for the experiments. Based on the measured and calculated results, a simple relationship was determined between *Bcr* and Re*, *f*, *R*, and *WR*, where the constants *K*_*1*_, *K*_*2*_, *K*_*3*_, and *K*_*4*_ depended on the physical properties of the melt and wall material:

$$Bcr\left({Re}^{*},f,R,WR\right)=\frac{{Re}^{*}}{{R}^{2}}({K}_{1} {f}^{-K2}+{K}_{3} {f}^{-K4} WR)$$

## Introduction

The microstructure of the solidified workpiece strongly depends on the melt flow, which evolves during the solidification. The cause of the melt flow might be the density difference between the different parts of the melt (buoyancy flow due to concentration and/or temperature difference) or stirring by magnetic induction (forced melt flow). The stirring by magnetic induction is extensively used at the solidification of different types of alloys and semiconductors, like the continuous casting of steels^1–3^. Both rotating magnetic field (RMF) and traveling magnetic field (TMF) can be implemented, but because the RMF facility is simpler, it is the most used technology.

During the last two decades, a lot of simulation was worked out to calculate the effect of forced melt flow on the solidified microstructure^4–13^. For the validating of the simulation, one of the most usual methods is to prepare some unidirectional experiments using low melting point alloys with well-known solidification parameters with RMF (temperature gradient, solid/liquid front velocity, magnetic induction, and frequency) and compare the calculated values with the simulated one.

One of the most problematic parts of these simulations is validating the calculated angular velocity of melt flow. It is known that it is impossible to obtain the flow information inside the casting via plant trials and directly from the laboratory solidification experiments using model alloys of low melting point like aluminium.

The melt flow induced by the magnetic field might be laminar or turbulent, depending on the angular velocity. The effect of the laminar and the turbulent melt flow on the microstructure is different, so it is important to know the flow type in the simulation at a given induction (*B)* that forms during solidification.

As mentioned before, the forced melt flow due to the RMF directly affects the solidified microstructure (primary and secondary dendrite arm spacing, micro and macro segregation, grain structure, columnar/equiaxed transition)^14–27^. Suppose the angular velocity is higher, the effect also higher. The surface friction due to wall roughness (WR) decreases the angular velocity, so this effect on the microstructure will be smaller at a given magnetic induction.

For the effect of the RMF, many examples can be found in the literature. Unfortunately, we did not find experiments where the authors gave information about the wall roughness. In one experiment series, the authors used only one type of crucible with a constant WR, and we did not find the same (similar) experiments series with different.

*The aim of these experiments is to show that if we want to compare the results of different experiments or the results of experiments with the results of simulations, it is substantial to determine the WR.*

In an earlier study^28^, we demonstrated that the critical magnetic induction (*Bcr*) at which the melt flow remains laminar or becomes turbulent depends on the frequency and diameter of the crucible during the rotating magnetic field (RMF) stirring of the melt. In that case, a TEFLON crucible was used during the experiments, the wall of the crucible was assumed to be sufficiently smooth (the wall roughness was negligible), and there was no friction between the wall and melt (Samples 1 – 8 in Table 1). Many different crucible materials were used in the solidification experiments to study the effect of magnetic stirring on the solidified microstructure, e.g., aerogel^15^, graphite^16–18^, Al_2_O_3_^19–23^, silica^24^, stainless steel^25,26^, gypsum^27^. The wall roughness of these crucibles was very different and was higher than that of TEFLON. The wall friction increased with an increase in wall roughness, and the angular velocity at a given magnetic induction (*B*) decreased. Therefore, *Bcr* was higher in the materials used in the solidification experiments^15–27^. The comparability of the different experiments requires knowledge of the effect of wall friction on the melt flow. Many studies [e.g.,^29–38^ have investigated wall friction, but we did not find any information about this effect in the case of magnetic stirring of the melt. *This study investigated this effect using extremely different wall roughness values. As the experiments were performed at room temperature, the Ga75In25 alloy was used from the usually used low melting temperature metals and alloys (Hg*^39^*, Ga *^40,41^*, GaIn alloy*^42^*, GaInSn alloy*^43,44^*) in the case of this type of investigations.*

**Table 1:** Physical parameters of the Ga75wt%In25wt% alloy.

	Ga75In25
Melting point, °C	15.7
Density, kg/m^3^ (at m.p.)	6517.5
Kinematic viscosity, m^2^/s	3.41 10^–7^
Specific electrical conductivity, MS/m	3.58
Relative magnetic permeability	1
Penetration distance**, at 50/100/150/200 Hz, mm	36/26/21/18

## Measuring methods

**Figure 1:**
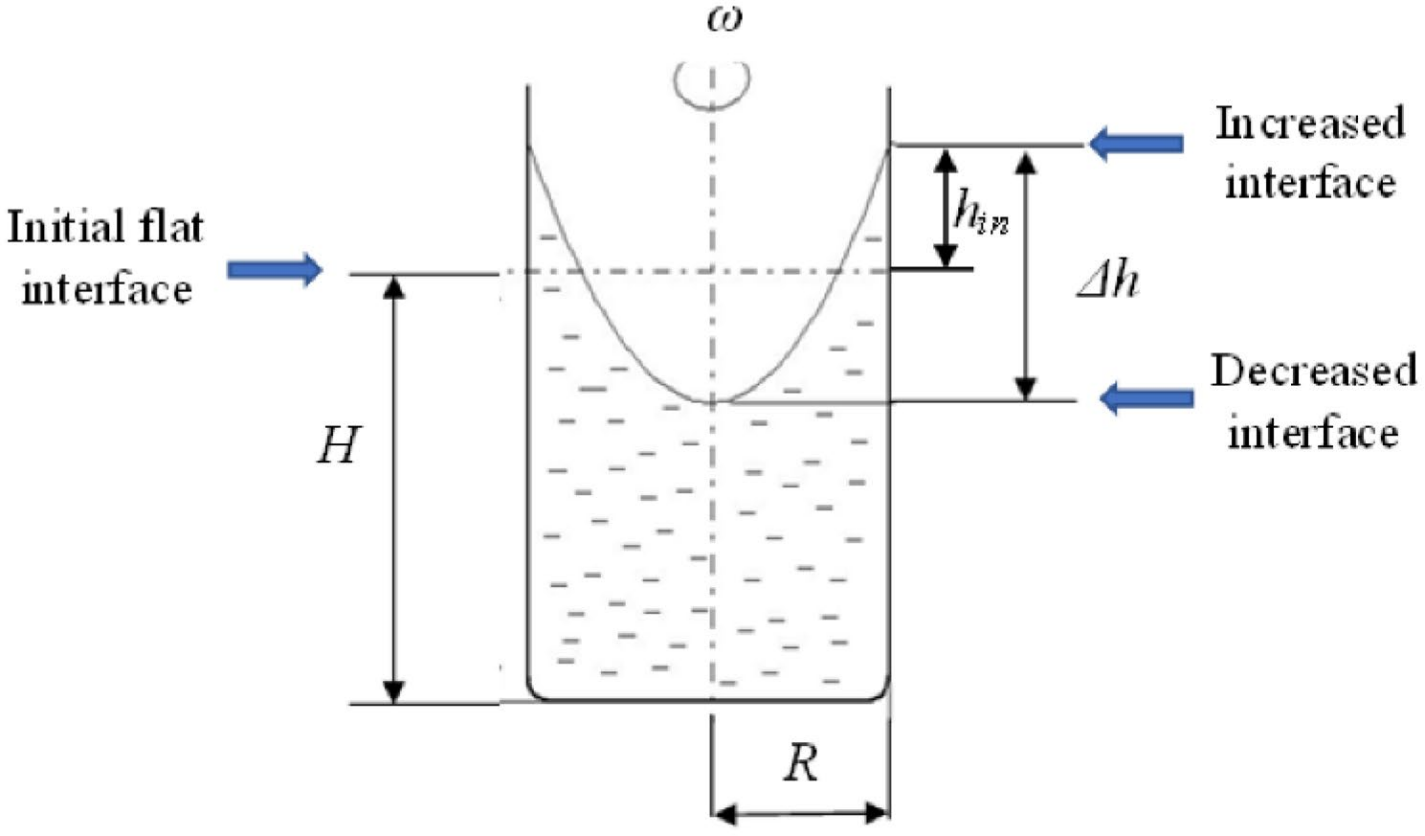
The effect of the rotating magnetic field (RMF) on the melt shape during stirring.

Pressure changes along the radius in a rotating liquid column as the liquid elements move at different velocities at positions with different radii. Consequently, a rotating paraboloid shape of the liquid surface develops in the case of a free surface (Fig. 1). There are two possibilities for determining the angular velocity of the metallic column developed by stirring the RMF.

(i) Measuring the change in the level of the free surface (height measuring method, HMM)^45^

The *Δh* = *2h*_*in*_ difference between the decrease at the centre and increase at the wall of the flat interface after a steady shape (Fig. 1) is obtained by calculating the angular velocity of rotation (see Eq. (1)). *H* is the height of the melt cylinder and position of the initial flat interface and *R* is its radius. In our experiments, the *H*/*R* aspect ratio changed between 60/5 = 12 and 60/12.5 = 4.8.

The angular velocity of the metallic column was calculated as follows:

1 $$\omega = \left(1/R\right){(2\Delta h g)}^{0.5}$$

**Figure 2:**
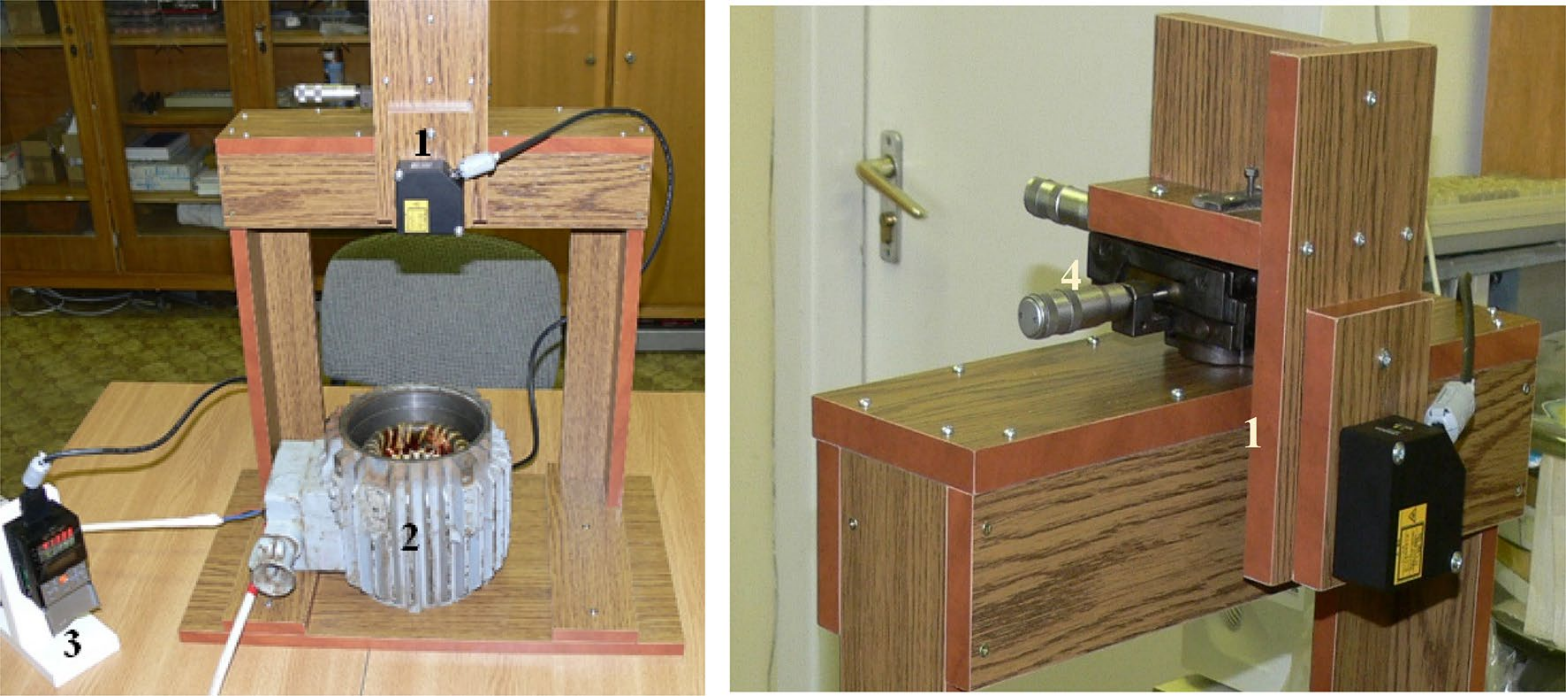
The unit for measuring the decrease in the level of the rotating metallic melt 1: laser, 2: inductor, and 3: display, 4:X – Y table.

A laser distance meter was used to measure the increase (*h*_*in*_) in the free surface of the metallic melt compared to the initial level, *H*, of the flat interface. The laser distance meter is illustrated in Fig. 2. The laser source (1 in Fig. 2) was fastened on an X -Y table. The laser was scanned on the bottom of the free surface, and the longest distance was accepted.

(ii) Measurement using the pressure compensation method (PCM)^46^

The pressure of the melt changes if the melt is rotated without a free surface, that is, in a closed tank. However, the pressure could be measured directly along the radius. A higher pressure corresponds to a larger radius. This phenomenon can be used to determine the average revolution number of the rotating melt stirred by an RMF. The pressure difference, *Δp*, related to the pressure prevailing at the axis of rotation can be calculated from the velocity differences of the melt elements that are present at any place with a radius of *r*. The peripheral speed was zero at the sample axis (*r* = *0*), whereas the maximum value was at the crucible wall (*r* = *R*).

**Figure 3:**
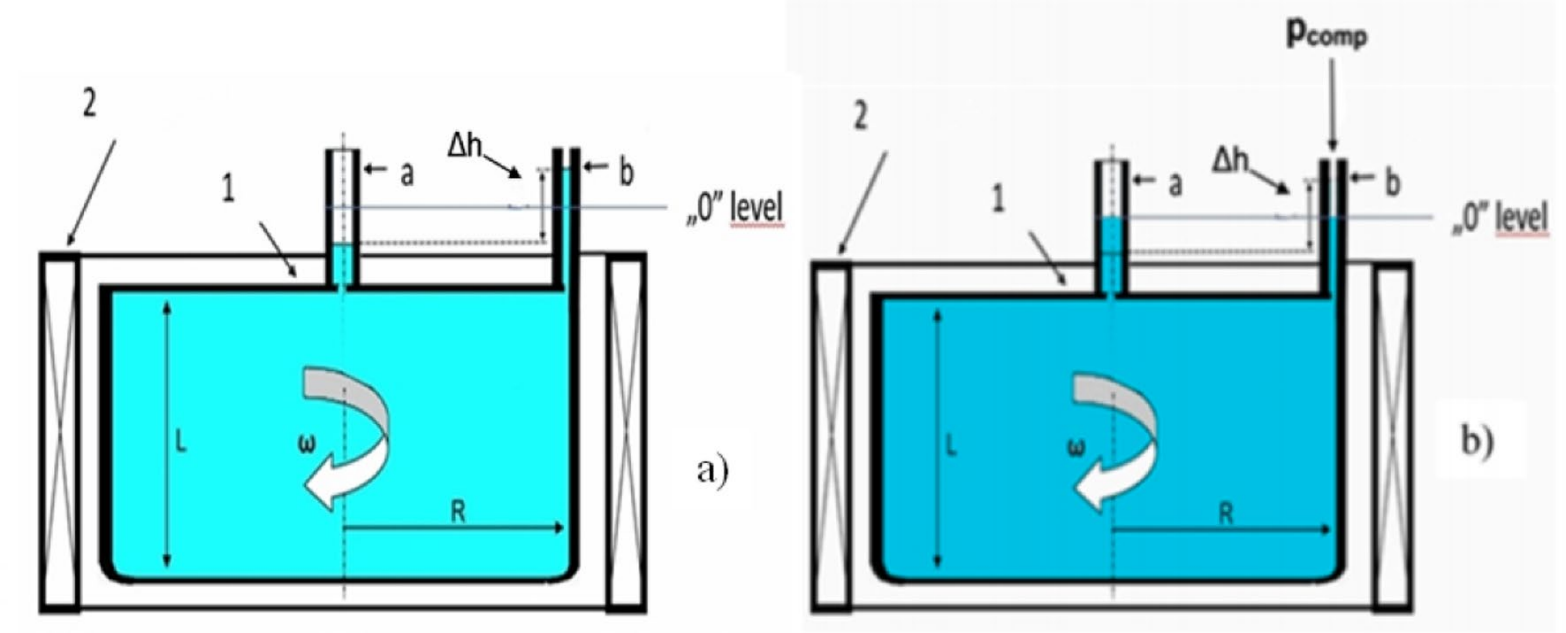
(**a**) The melt level in the gauges when the magnetic induction was occurring (1) closed tank, (2) rotating magnetic field (RMF) inductor, and ‘a’ and ‘b’ gauges. (**b**) The melt level in the gauges at the pressure compensation.

Measuring the pressure developing in the melt in the closed probe was difficult without disturbing the melt flow; therefore, the pressure was not measured directly in the closed probe. To perform the pressure measurement at *r* = *R*, the tank was closed with two gauges connections. The gauges at the axis (*r* = *0*) and periphery (*R*) of the tank are labeled respectively ‘a’ and ‘b’ in Fig. 3a. The two gauges connections and tank were a ‘communication vessel.’ The melt level was the same at the gauge connections if the RMF inductor did not operate. Moreover, the atmospheric pressure was identical in the gauge connections, the so-called ‘stationary-level’ or ‘0-level’. As shown earlier^1^, the *r* = *R − *0.2 mm position was chosen to measure the pressure because of the maximum pressure difference. Therefore, the relative error of the measured pressure was minimised.

A level difference of *Δh* developed between the melt levels in the ‘a’ and ‘b’ gauge connections when the melt was rotated (stirred) by the RMF inductor (Fig. 3b). The *ρgΔh* metal-static pressure of the melt column was in equilibrium with the pressure difference (*Δp*) that developed between the axis and periphery of the tank. If the free surface of the melt is at atmospheric pressure in the gauge connections, that is

2 $$\Delta {p}_{max}=\frac{\rho [{v(R)]}^{2}}{2}=\frac{\rho {\omega }^{2}{R}^{2}}{2}=\rho g\Delta h$$

The angular velocity (*ω*) of the metallic column can be calculated as follows:

3 $$\omega =\frac{1}{R}{(2\Delta p/\rho )}^{0.5}$$

To determine the pressure difference, the melt surface was returned to the ‘0’ level of the two gauges connections by supplying air at pressure *P*_*comp*_ of the ‘b’ gauge with the pneumatic system. The accuracy of the pressure measurements was 20 Pa. As the measured pressure was higher than 20 Pa in most cases, this accuracy was sufficient. Details of the measurement and equipment are described elsewhere^12^.

## Experiments

The Ga75wt%In25wt% alloy was chosen because the experiments were performed at room temperature. The physical parameters of the alloys are listed in Table 1. Two sets of experiments were performed (Table 2). Column 3 in Table 2 shows the methods used for the samples.

**Table 2:** The characterisation of the samples and the measured and calculated critical magnetic induction values (*Bcr*) using the pressure compensation method (PCM) and height measuring method (HMM).

No. Sample	Wall material	Measuring method	*WR* mm	*R* mm	*f* Hz	Re* = 2320		Re* = 4000	
						*Bcr(meas)*	*Bcr(calc)*	*Bcr(meas)*	*Bcr(calc)*
1	TEFLON	PCM	0.04	5	50	17.71	17.66	30.53	30.44
2	TEFLON	PCM	0.04	7.5	50	7.30	7.85	12.58	13.53
3	TEFLON	PCM	0.04	12.5	50	2.50	2.82	4.31	4.87
4	TEFLON	PCM	0.04	5	150	7.71	8.86	13.29	15.28
5	TEFLON	PCM	0.04	7.5	150	3.13	3.94	5.39	6.79
6	TEFLON	PCM	0.04	12.5	150	1.03	1.42	1.78	2.45
7	TEFLON	PCM	0.04	5	100	11.26	11.42	19.42	19.68
8	TEFLON	PCM	0.04	5	200	7.03	7.41	12.12	12.78
9	TEFLON	PCM	0.04	5	150	7.74	8.78	13.46	15.28
10	ALOX1	PCM	0.082	5	150	8.8	9.98	15.31	17.36
11	ALOX2	PCM	0.107	5	150	9.53	10.69	16.58	18.60
12	P40	PCM	0.275	5	150	14.76	15.48	25.68	26.92
13	TEFLON	PCM	0.04	5	50	17.73	17.35	30.84	30.16
14	ALOX1	PCM	0.082	5	50	19.00	18.98	33.10	32.99
15	ALOX2	PCM	0.107	5	50	19.36	19.95	33.67	34.68
16	P40	PCM	0.275	5	50	27.48	26.46	47.79	46.00
17	Oil Glass	HMM	0.00	5	150	6.96	7.64	12.11	13.30
18	Dry Glass	HMM	0.15	5	150	7.10	8.07	12.35	14.04
19	P150	HMM	0.155	5	150	8.88	12.06	15.44	20.98
20	P100	HMM	0.165	5	150	11.22	12.35	19.51	21.47
21	P60	HMM	0.209	5	150	12.54	13.60	21.81	23.65
22	P40	HMM	0.275	5	150	14.8	15.48	25.74	26.92

**Figure 4:**
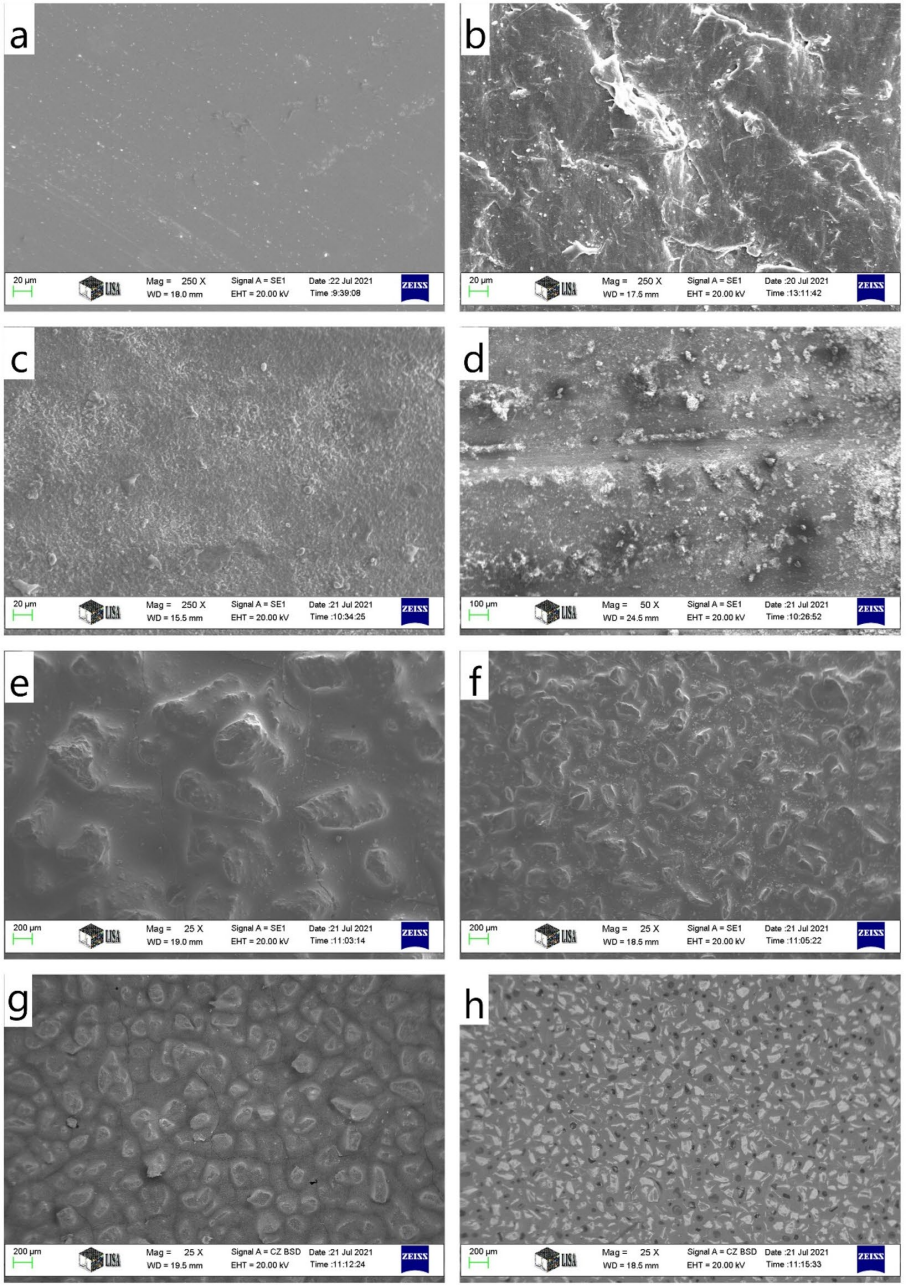
The surface of the used crucible materials, scanning electron microscopy (SEM) secondary electron (S.E.) images (**a**): glass, (**b**): TEFLON, (**c**): fine ALOX, (**d**): rough ALOX, (**e**): P40, (**f**): P80, (**g**): P120, and (**h**): P150.

(i) Molten alloy was placed in a crucible (glass sample holder) with an internal diameter of 13 mm and a height of 100 mm. The height of the molten alloy ‘melt cylinder’ was 60 mm. The induction of the magnetic field was 72 mT, and the angular velocity of its rotation was 942 rad/s (the pole number of the three-phase inductor was two, and the frequency of its power-supply voltage was 150 Hz). During these experiments, six different values of the wall surface roughness (*WR*) were used on the internal surface of the sample holders. These different roughness values were achieved with oiled glass, dry glass, and glass covered with abrasive papers with a roughness corresponding to P150, P100, P60, and P40. The “PX” is the commercial mark of the abrasive paper (P means: paper). The difference between the abrasive papers is the diameter of the corundum (Al_2_O_3_) particles. The average diameter is 450, 280, 140, and 90 µm for the P40, P60, P100, and P150, respectively. The laser distance meter measured the value of WR of the different crucible walls was also used to measure the decrease (Δh) in the free surface of the metallic melt. Each 50th µm of the length of 3000 µm was measured. The average of the 60 measured values was the “0” line. After that, the average of the higher and lower length from the value of the “0” line was calculated. WR was characterised by the difference between these two average values. The values obtained in this manner are presented in Table 1. The oiled glass was considered to have a smooth surface, that is, its *WR* was 0 µm. The surface of the samples was investigated by SEM. Before the investigation, the samples were evaporated by Au, because the electrical conductivity of the samples is negligible. The SEM images were produced by secondary electrons (SE). The differences between the surfaces of the samples are shown in Fig. 4.

The thickness of the applied abrasive paper decreased the inside diameter of the sample holder by 3 mm (the thickness of the abrasive paper was approximately 1 mm, and the vent between the crucible wall and the abrasive paper was approximately 0.5 mm). The effective diameter of the crucible was 10 mm^45^.

(ii) During the experiments, melt cylinders with radii of 5, 7.5, and 12.5 mm at frequencies of 50, 100, 150, and 200 Hz in a magnetic induction range of 0 – 90 were used. The height of the melt cylinder (*H*) was 100 mm. The radius of the melt cylinder (*R*) was smaller than the penetration distance in each case, except the case of 200 Hz and *R* = 12.5 mm. The crucible material was TEFLON^1^, ALOX, and the abrasive paper P40^46^. The *H*/*R* ratio changed between 100/5 = 20 and 100/12.5 = 8, thus, the influences of the penetration distance and ‘end effect’ on the measurement results were neglected.

## Results and discussion

**Figure 5:**
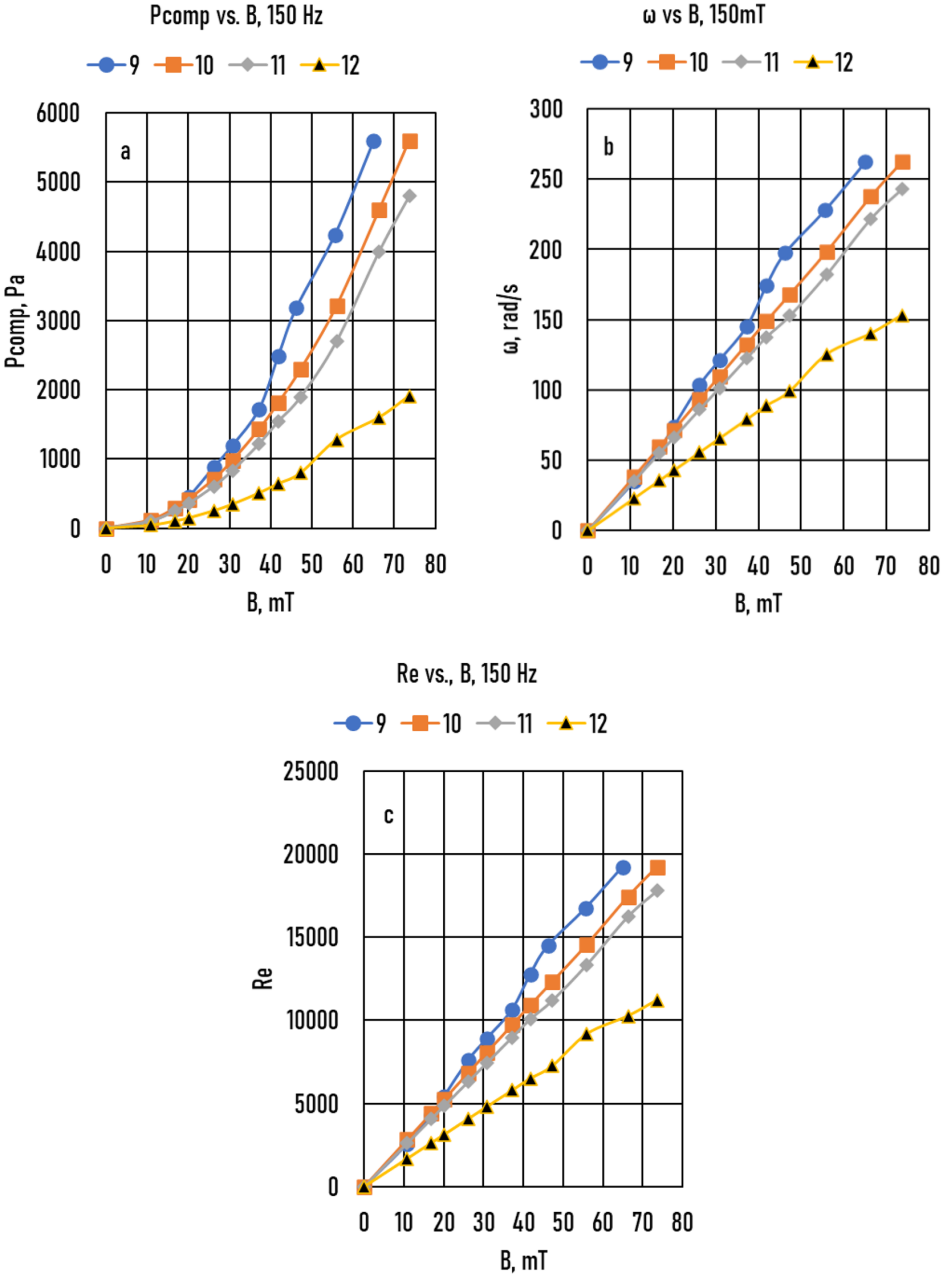
Samples 9 – 12 (**a**) Measured compensation pressure (*P*_*comp*_) as a function of magnetic induction (*B*). (**b**) Angular frequency of melt (*ω*) as a function of *B*. (**c**) The real Reynolds number (*Re*) as a function of *B*.

**Figure 6:**
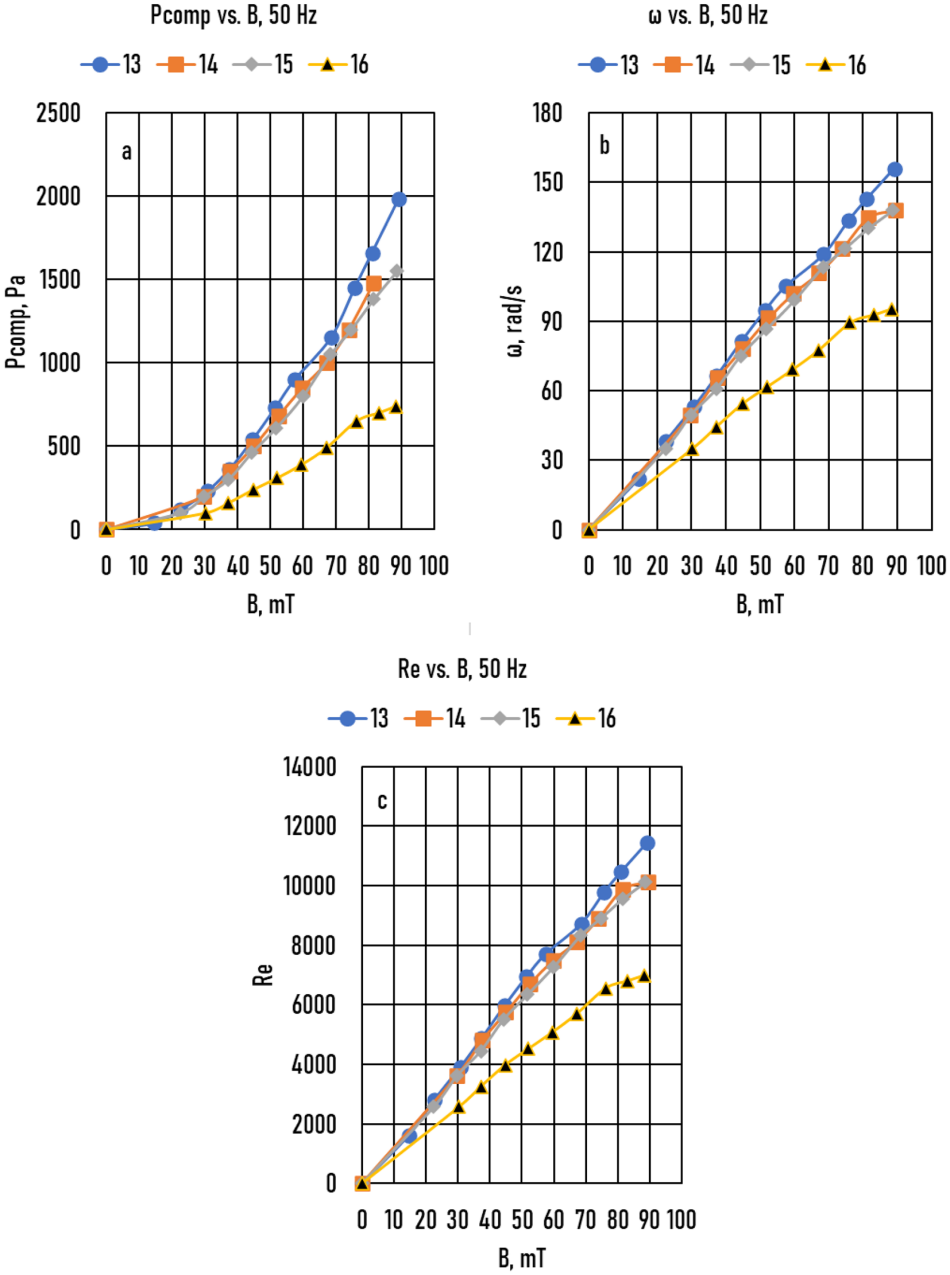
Samples 13 – 16 (**a**) Measured compensation pressure (*P*_*comp*_) as a function of magnetic induction (*B*). (**b**) Angular frequency of melt (*ω*) as a function of *B*. (**c**) The real Reynolds number (*Re*) as a function of *B*.

**Figure 7:**
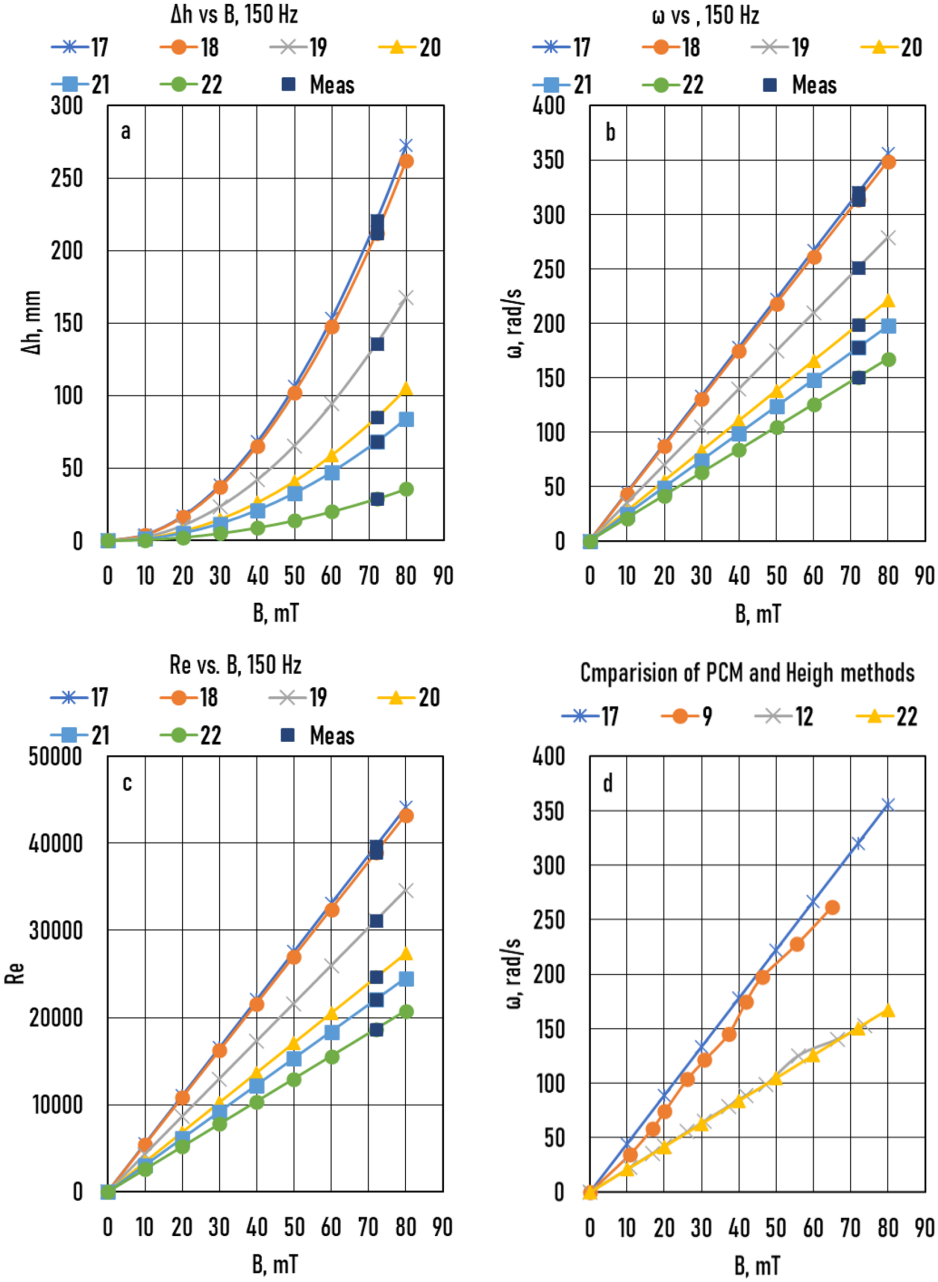
Samples 17 – 22 (**a**) Measured height (*Δh*) as a function of magnetic induction (*B*). (**b**) Angular frequency of melt (*ω*) as a function of *B*. (**c**) The real Reynolds number (*Re*) as a function of *B*. (**d**) Comparison of the pressure compensation method (PCM) and height measuring method (HMM).

The results of the measured *P*_*comp*_*(B)* functions are shown in Fig. 5a (Samples 9 – 12) and Fig. 6a (Samples 13 – 16), and the measured *Δh(B)* function is shown in Fig. 7a (Samples 17 – 22). The angular frequency was calculated using Eq. 3 for Samples 9 – 12 (Fig. 5b) and Samples 13 – 16 (Fig. 6b). For Samples 17 – 22, only one *Δh(B)* point was measured at *B* = 72 mT (a big black point in the figure). From this data, *ω*(*B* = 72 mT) was calculated using Eq. 1, and the *ω*(*B*) function was calculated from *B* = 0 *to B* = 90 mT because the function is a straight line at *B* = 0 and *ω* = 0 (Fig. 7b). The *Δh*(*B*) function was calculated from these data. The angular velocities calculated using the PCM and HMM are compared in Fig. 7d. In the cases of Sample 17 (HMM) and Sample 9 (PCM method), the sample radius (*R*) and frequency of the magnetic induction (*f*) were the same (5 mm and 150 Hz), whereas the value of *WR* of Sample 9 was slightly higher than that of Sample 17 (0.04 and 0.0). As a result of this small difference, the values of the *ω*(*B*) function of Sample 9 were slightly smaller than those of Sample 17. In the cases of Sample 12 (PCM) and Sample 22 (HMM), *R*, *f*, and *WR* were the same, and the values of the two *ω*(*B*) functions were also the same. The main difference between the two methods was the friction between the melt and closing plate of the tank in the PCM. Comparing the results of the two methods, the effect was negligible.

Using the *ω*(*B*) functions, the Reynolds number *(Re)* as a function of magnetic induction (*Re*(*B*)) functions was calculated (Samples 9 – 12, Fig. 5c; Samples 13 – 16, Fig. 6c; and Samples 17 – 22, Fig. 7c):

4 $$Re= \omega {R}^{2}/\nu$$

where *ν* = 3.41 10^−7^ m^2^/s is the kinematic viscosity of the Ga75In25 alloy.

As the *Re*(*B*) function is a straight line in the investigated regime of magnetic induction (0 mT < B < 90 mT), the equation of this function is:

5 $$Re=m B$$

where *m* is the slope of the *Re*(*B*) function.

The *Bcr* values at which the flow in the melted alloy changed from laminar to unstable (at Re* = *2320*) and from unstable to turbulent (at Re* = 4000)^47–49^ were determined using Eq. (6):

6 $$Bcr= {Re}^{*}/m$$

The *Re*(*B*) function and *Bcr* (Re* = 2320, Re* = 4000) values of Samples 1 – 8 were determined in an earlier study^28^. The calculated *Bcr calc* values using Eq. (6) are shown in columns 8 (Re* = 2320) and 10 (Re* = 4000) of Table 2. In this case, *WR* was 0.04 mm.

**Figure 8:**
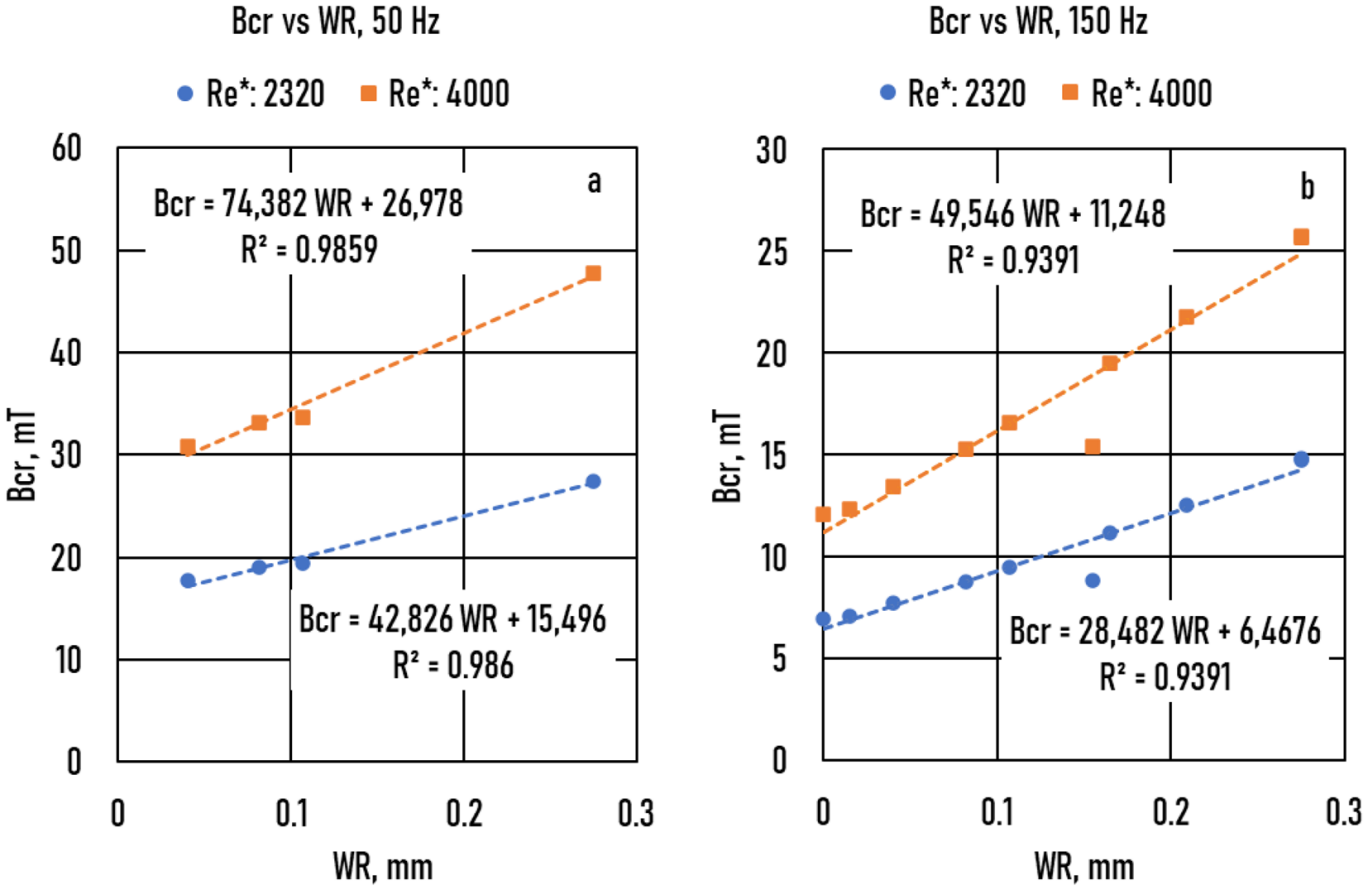
The critical magnetic induction (*Bcr*) as a function of wall roughness (*WR*) for real Reynolds numbers (Re*), (**a**): *f* = 50 Hz, (**b**): *f* = 150 Hz.

The *Bcr* values of the samples with *R* = 5 mm are shown as a WR function in Figs. 8a (*f* = 50 Hz) and Fig. 8b (*f* = 150 Hz). The *Bc*r*(WR*) functions are straight lines.

7.a $$Bcr(R=5mm,WR{, Re}^{*},f)= Bcr\left( R=5 mm, {WR=0, Re}^{*}, f\right)+ \Delta Bcr\left(R=5 mm, WR, {Re}^{*}, f\right)$$

**Figure 9:**
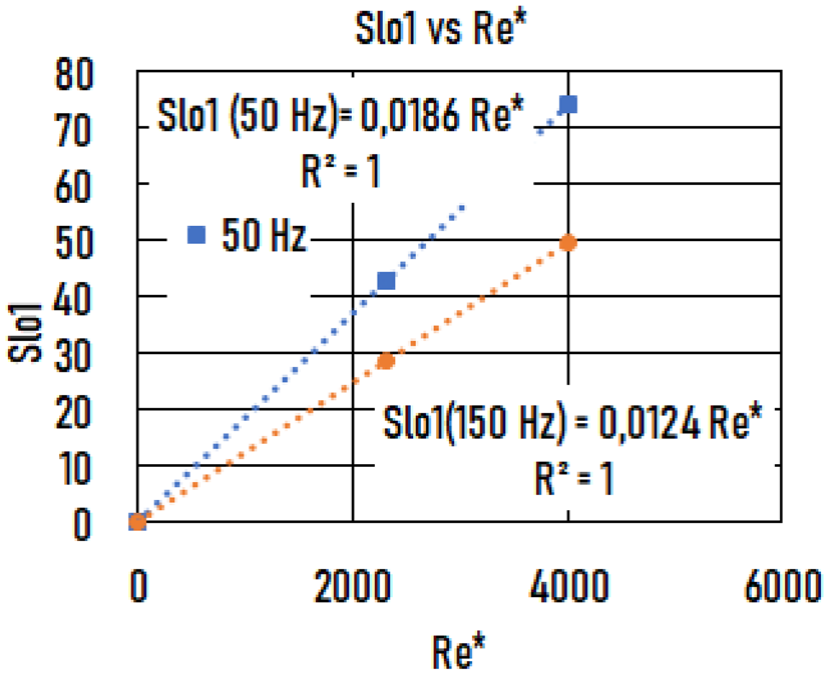
The slope (*Slo1*) of the critical magnetic induction (*Bcr* (wall roughness (*WR*))) function vs. Reynolds number (Re^*^).

**Figure 10:**
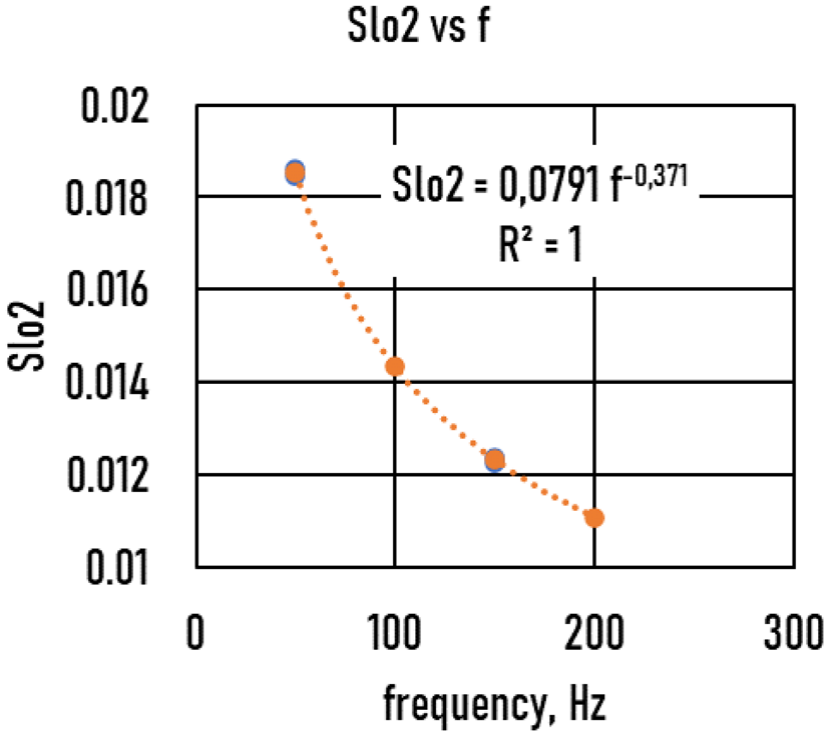
The slope (*Slo2*) of *Slo1*(Re*) function vs. frequency (*f*).

The slope of the *Bcr*(*WR*) function, *Slo1,* as a function of Re^*^ is shown in Fig. 9. The Slo1(Re*) functions are again straight lines and depend on frequency. The slope of the *Slo1*(Re*) function, *Slo2*, is shown in Fig. 10. Based on these functions, the Δ*Bcr* (*R* = 5 mm, Re^*^, f, WR) function is

7.b $$\Delta Bcr\left(R=5mm, WR, {Re}^{*},f\right)=\mathrm{0,0791}{Re}^{*} {f}^{-0.37}WR$$

The *ΔBcr* value at a given R:

7.c $$\Delta Bcr\left(R, WR, {Re}^{*},f,\right)=0.0791{Re}^{*}{f}^{-0.37}WR(\frac{{5}^{2}}{{R}^{2}})=\mathrm{1,97}{Re}^{*}\frac{{f}^{-0.37}WR}{{R}^{2}}$$

*Bcr*(R, f, Re*, WR = 0) can be calculated from the measured value of *Bcr*(*meas*)*:*

8 $$Bcr\left(R, f, {Re}^{*}, WR=0\right)=Bcr\left(meas\right)- 1.97{Re}^{*}\frac{{f}^{-0.37}WR}{{R}^{2}}$$

The type of the $Bcr(R, f, Re*, WR=0)$ function is described in our earlier paper^28^:

9 $$Bcr\left(R, f, {Re}^{*}, WR=0\right)={K}_{1}{Re}^{*}{f}^{-n}/{R}^{2}$$

Rearranging (Eq. 9):

10 $$Y=Bcr\left(R, f, {Re}^{*}, WR=0\right){R}^{2}/{Re}^{*}={K}_{1}{f}^{-n}$$

**Figure 11:**
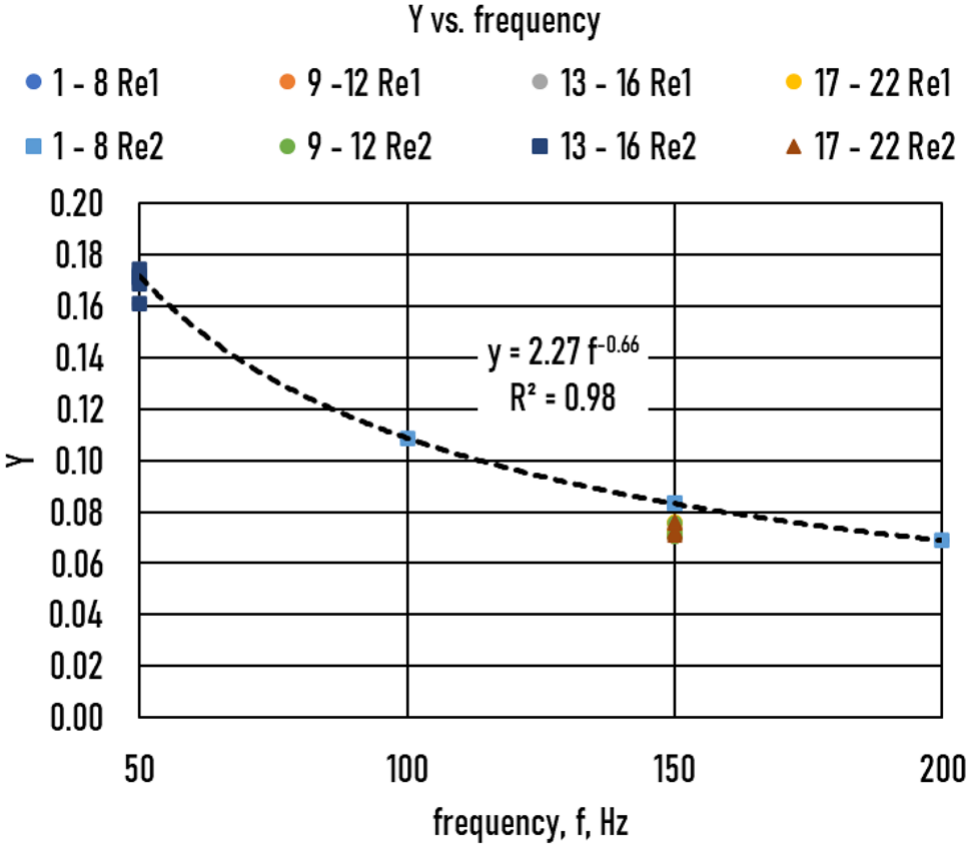
Y vs. frequency of all the samples. Reynolds number (*Re1* = 3220, *Re2* = 4000).

Using the calculated values of *Bcr* (Re*, f, R, WR = 0) for all samples, this function is shown in Fig. 11. Based on the trendline:

11 $$Y=2.27{f}^{-0.66}$$

and then

12 $$Bcr\left({Re}^{*},f,R,RW=0\right)=2.27{Re}^{*}{f}^{-0.66}/{R}^{2}=A{f}^{-n}/{R}^{2}$$

This equation differs slightly from Eq. (12) in^28^, where neither A nor *n* depends on Re* (*n* =  − 0.628 at Re* = 2320 and *n* =  − 0.695 at Re* = 4000).

Finally, the critical magnetic induction (*Bcr)* at a given Reynolds number (Re*) as a function of the sample radius (*R*)*,* frequency (*f*)*,* and wall roughness (*WR*) was calculated as follows:

13 $$Bcr\left({Re}^{*},f,R,RW\right)=\frac{{Re}^{*}}{{R}^{2}}(2.27 {f}^{-0.66}+1.97 {f}^{-0.37} WR)$$

In the case of a very smooth crucible (*WR* = *0*), such as oiled glass, the laminar/unstable transition is:

14 $$Bcr=5266\frac{{f}^{-0.66}}{{R}^{2}}$$

and the unstable/turbulent transition is:

15 $$Bcr=9080\frac{{f}^{-0.66}}{{R}^{2}}$$

In the case of *WR* > 0, the laminar/unstable transition is:

16 $$Bcr\left({Re}^{*}=2320,f,R,RW\right)=\frac{1}{{R}^{2}}(5266 {f}^{-0.66}+4570 {f}^{-0.37} WR)$$

and the unstable/turbulent transition is:

17 $$Bcr\left({Re}^{*}=4000,f,R,RW\right)=\frac{1}{{R}^{2}}(9080 {f}^{-0.66}+7880 {f}^{-0.37} WR)$$

**Figure 12:**
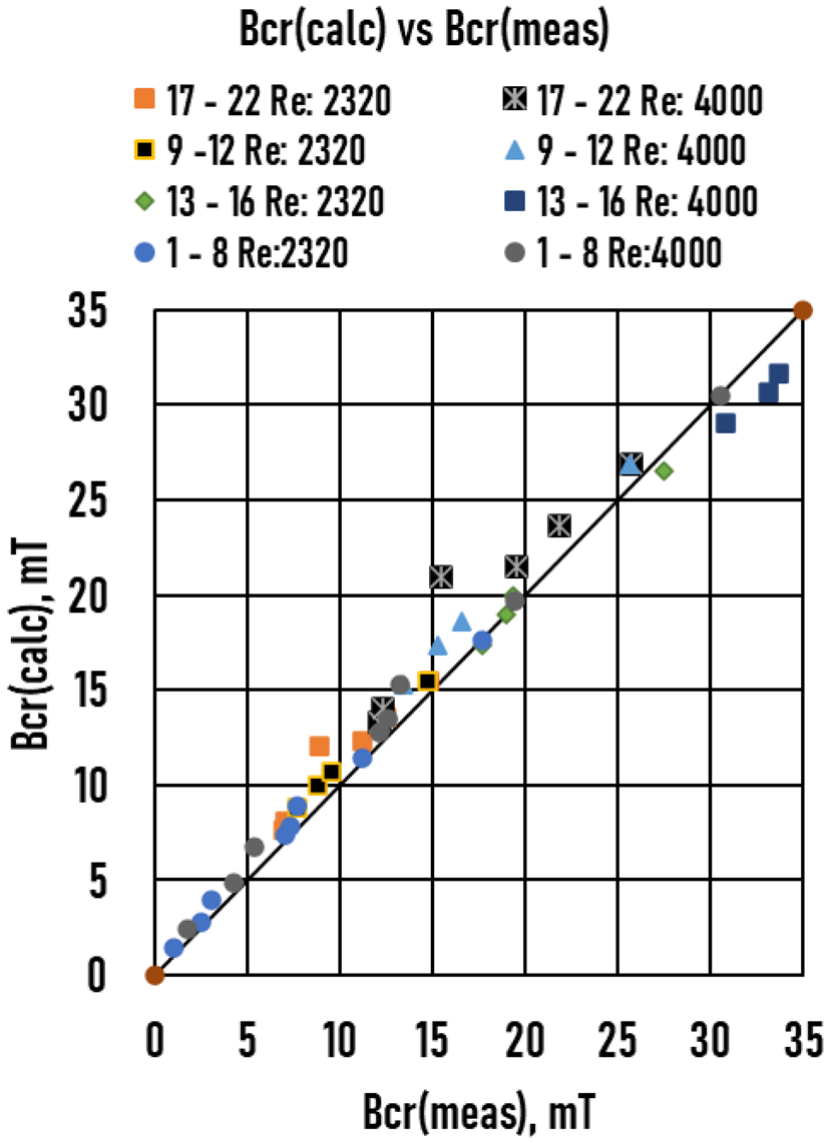
Comparison of the calculated (Calc. Bcr) and measured (Meas. Bcr) critical magnetic induction.

The measured (*Bcr meas*) and calculated (*Bcr calc*) values are compared in Fig. 12 and Table 2. For all 22 samples, R^2^ = 0.99; therefore, the agreement was suitable.

**Figure 13:**
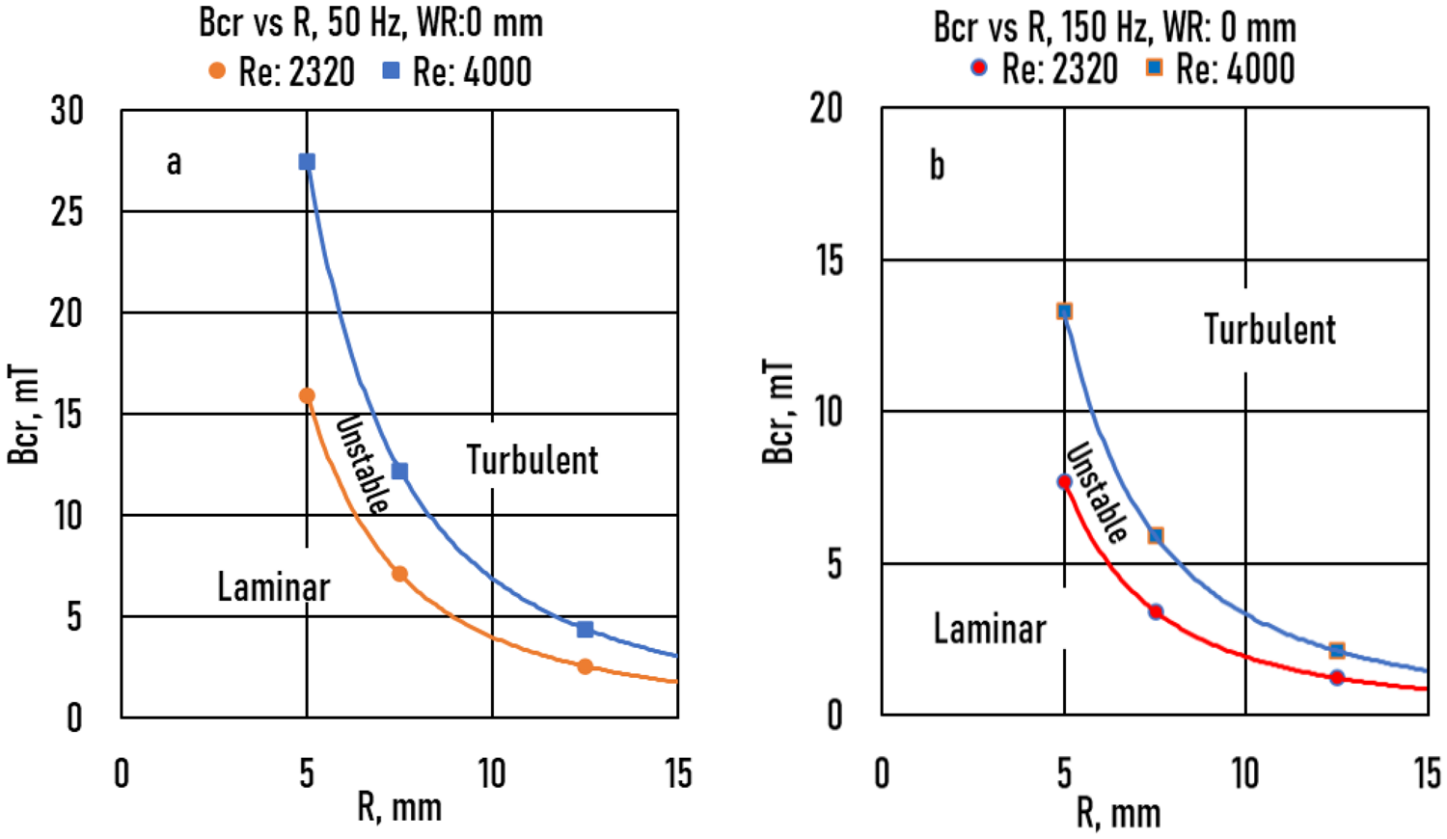
The critical magnetic induction (*Bcr*) as a function of sample radius (*R*) (**a**): *f* = 50 Hz, (**b**): *f* = 150 Hz at *WR* = 0.

Using Eqs. (16 and 17), two plots were constructed to show the laminar/unstable and unstable/turbulent transitions at 50 and 150 Hz for *WR* = 0 (Fig. 13). These plots shown in Fig. 8 were similar to those in a previous study^28^. In this study, the differences considered the wall roughness of TEFLON (*WR* = 0.04 mm). The unstable range was very narrow at both 50 and 150 Hz.

**Figure 14:**
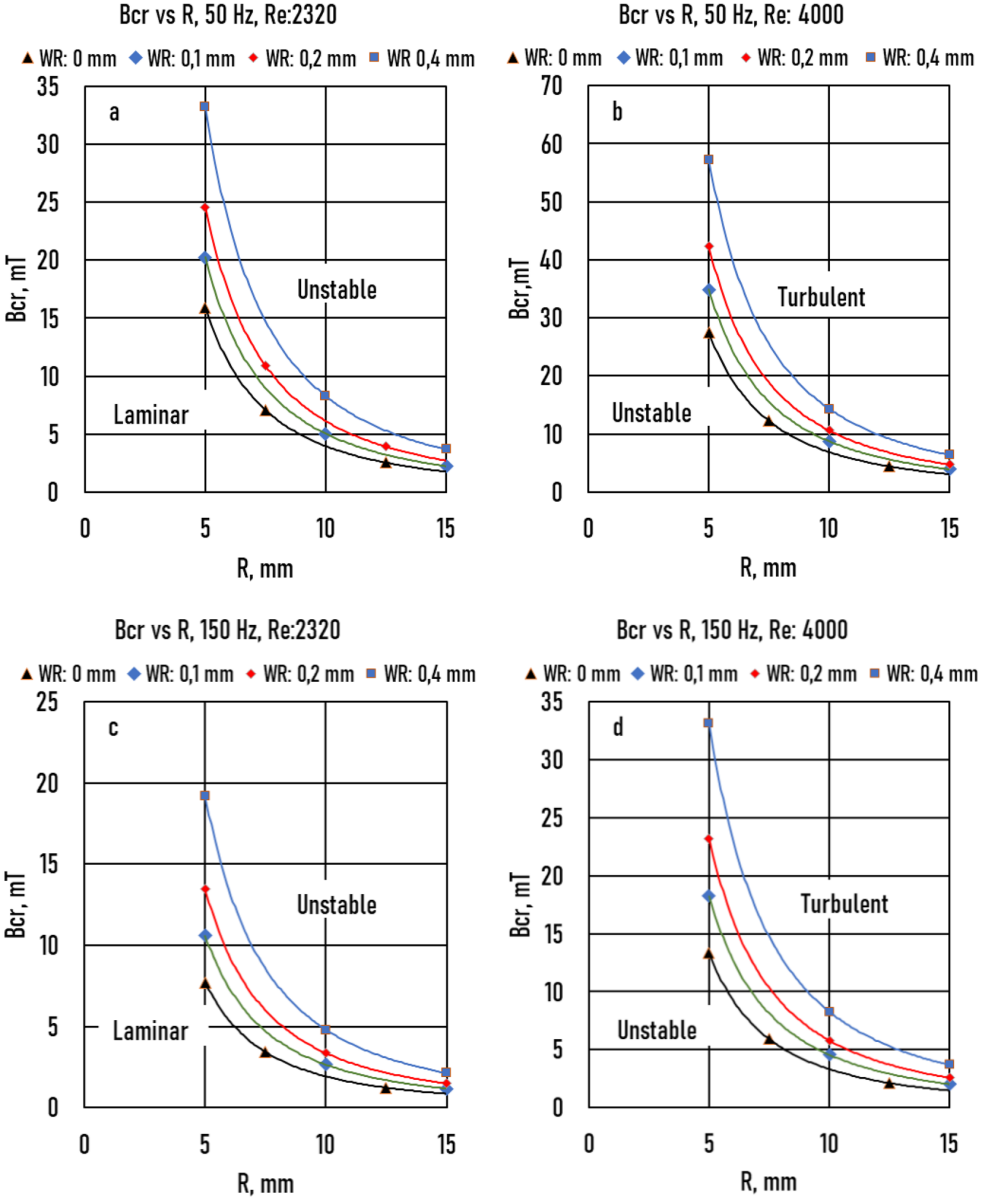
Effect of Wall Roughness (*WR*) on the critical magnetic induction (*Bcr*) (**a**): *f* = 50 Hz, *Re* = 2320; (**b**): *f* = 150 Hz, *Re* = 4000; (**c**): *f* = 150 Hz, *Re* = 2320; (**d**): *f* = 150 Hz, *Re* = 4000.

In Fig. 14, the effect of wall roughness was demonstrated on the transients in the cases of 50 and 150 Hz. Wall roughness had a significant effect on transients.

Vortexes may have developed near the wall of the crucible, which can result in unstable or turbulent flow at a lower *Bcr* value than the calculated one. With the two measuring methods, this effect was not demonstrated; this method probably does not currently exist. Numerical simulation is the only method capable of studying this effect. In the case of the TEFLON crucible (WR = 0.04 mm), with numerical simulation, it was verified that near the crucible wall, the turbulent flow at a given magnetic induction while inside the flow remains laminar^50^.

Equations (13 – 17) are valid only for the Ga75In25 alloy, a maximum 12.5 mm radius, and a cylindrical sample. If the alloy, shape of the sample (e.g., rectangular), or the maximum radius is changed, the type of the $Bcr(R, f, {Re}^{*}, WR)$ function will be the same, but the constants will change.

## Summary

In a previous study^1^, the PCM was used. We showed that the angular frequency of the melt was significantly smaller than that of magnetic induction with RMF stirring. In this case, a TEFLON crucible with a relatively smooth surface was used during the experiments. The wall roughness can significantly change the wall friction and angular velocity of the melt flow. In practical solidification experiments, the wall roughness of the crucible material differed (rougher) from that of TEFLON. We must know the effect of the wall roughness to compare the different experiments and validate the melt flow simulations.

To obtain information about the effect of the wall roughness, we determined the angular frequency of the melt as a function of the magnetic induction (*B*) and the frequency (*f*) of the rotating magnetic field using two different methods (PCM and HMM). The crucible materials used were oiled and dry glass, two types of ALOX, TEFLON, and glass covered with abrasive papers with roughness corresponding to P150, P100, P60, and P40. The wall roughness, measured using a laser distance meter, was characterised by the average difference between the measured minimum and maximum distances. Based on the calculated *Re* number as a function of magnetic induction considering the wall roughness, we determined the magnetic inductions at which the flow changed from laminar to unstable and from unstable to turbulent.

## Conclusion

(i) Two measurement methods (PCM and HMM) were used to determine the angular velocity of the melt. The measured angular frequencies were practically the same using the same experimental conditions for the two measuring methods (same magnetic induction and frequency, diameter, and crucible material).

(ii) The magnetic induction belonging to the laminar/unstable and unstable/turbulent transients (*Bcr*) were calculated using one equation as a function of the frequency of the magnetic field (f), critical Reynolds number (Re*), radius of the crucible (*R*), and roughness of the crucible wall (*WR* ):

18 $$Bcr\left({Re}^{*},f,R,WR\right)=\frac{{Re}^{*}}{{R}^{2}}({K}_{1} {f}^{-K2}+{K}_{3} {f}^{-K4} WR)$$

where *K1*, *K2*, *K3*, and *K4* are constants that depend on the physical properties of the melt (density, electrical conductivity, and kinematic viscosity).

(iii) If the wall roughness increases and Bcr belonging to the transients increases significantly, different experiments or melt flow simulations must be validated and performed.

(iv) The effect of the wall friction of TEFLON was minimal (*WR* = 0.04), as was previously proposed^1^.

(v) The wall roughness had a significant effect on the transients.

(vi) The unstable range was very narrow at 50 and 150 Hz.

(vii) Equations (13–17) were valid only for the Ga75In25 alloy, a maximum 12.5 mm radius, and a cylindrical sample. If the alloy, shape of the sample (e.g., rectangular), or radius changes, the type of the $Bcr(R, f, {Re}^{*}, WR)$ function will be the same, but the constants will change.

## Data Availability

The datasets used and analysed during the current study available from the corresponding author on reasonable request.

## Abbreviations

*r*: The radius at a given point in the melt cylinder, mm
*R*: The radius of the melt cylinder, mm
*H*: The height of the melt cylinder, mm
*Δ**p*_*max*_: The maximum pressure at r = R, Pa
*ρ*: Density of the melt, kg/m^3^
*ω*: Angular velocity of rotation (rad/s)
*g*: Gravitational constant, 9.81 m^2^/s
*h*_*in*_: Increase of the free surface at r = R, mm
*Δh*: *the* Difference between the decrease at the centre and increase at the wall of the flat interface, mm
*f*: The frequency of the RMF, Hz
*WR*: Wall roughness, mm
*Re*: The Reynolds number
*Re**: Critical Reynolds number at laminar/unstable (2320) and unstable/turbulent transition (4000).
*RMF*: Rotating magnetic field
*B*: Magnetic induction of RMF, mT
*Bcr(meas)*: Measured critical magnetic induction at laminar/unstable and unstable/turbulent transitions, mT
*Bcr(calc)*: Calculated critical magnetic induction at laminar/unstable and unstable/turbulent transitions, mT
*ΔBcr*: Contribution of wall roughness to critical magnetic induction, mT
*K2*, *K3*, *K4*, and *K5*: Are constants and depend on the physical constants of the melt (density, electrical conductivity, kinematic viscosity)
